# Bitter Chinese Herbal Medicine Exerts Pharmacological Effects via TAS2Rs: A Systematic Review from Natural Ligands to Therapeutic Potential

**DOI:** 10.3390/ijms27073073

**Published:** 2026-03-27

**Authors:** Lian Li, Ruitong Dong, Shibu Feng, Yan Huang, Xin Li, Hanyun Que, Huan Li, Peng Wang, Leu-Kim Fey, Yi Zhang, Zhaotong Cong, Sanyin Zhang

**Affiliations:** 1 Innovative Institute of Chinese Medicine and Pharmacy, Chengdu University of Traditional Chinese Medicine, Chengdu 611137, China; ll1973163811@163.com (L.L.); ruitong_dong@163.com (R.D.);; 2 School of Preclinical Medicine, Chengdu University of Traditional Chinese Medicine, Chengdu 611137, China; 3 School of Clinical Medicine, Chengdu University of Traditional Chinese Medicine, Chengdu 611137, China; 4 School of Ethnic Medicine, Chengdu University of Traditional Chinese Medicine, Chengdu 611137, China; 5 Institute of Herbgenomics, Chengdu University of Traditional Chinese Medicine, Chengdu 611137, China; 6 School of Pharmacy, Nanjing University of Chinese Medicine, Nanjing 210023, China; 7 School of Pharmacy, Chengdu University of Traditional Chinese Medicine, Chengdu 611137, China; 8 M Kandiah Faculty of Medicine and Health Sciences, Universiti Tunku Abdul Rahman, Kajang 43000, Malaysia

**Keywords:** Chinese herbal medicine, bitter taste receptors, pharmacological effects, signaling pathway

## Abstract

Bitter compounds may function not only as taste substances but also as important active constituents mediating therapeutic effects. Their recognition is primarily mediated by bitter taste receptors (TAS2Rs), which exert pharmacological effects, such as regulating glucose metabolism, anti-inflammatory properties, and immune modulation, aligning closely with the therapeutic effects of bitter Chinese herbal medicine (BCHM). Contemporary pharmacological research has increasingly underscored the therapeutic potential of bitter traditional Chinese medicine (TCM), particularly through their bioactive constituents in the prevention and treatment of diverse pathological conditions. Here, we systematically review the diversity of bitter compounds from TCM and features of TAS2Rs, including their tissue distribution, physiological functions, structural characteristics, signal transduction mechanisms, and single-nucleotide polymorphisms. While numerous bitter phytochemicals have been characterized as agonists of TAS2Rs, the precise physiological functions and underlying molecular mechanisms mediated by TAS2R activation remain incompletely elucidated. This knowledge gap is largely attributable to several methodological and biological challenges, including the widespread tissue distribution of TAS2Rs, the complexity of their downstream signaling cascades, and the structural and functional heterogeneity of bitter compounds. This review outlines theoretical foundations, future perspectives and challenges for the drug development of TAS2R from BCHM.

## 1. Introduction

In traditional Chinese medicine (TCM) theory, bitter is one of the “Five Flavors.” It is not only a fundamental taste sensation but also a highly systematic generalization of the efficacy and action patterns of medicinal substances. It possesses multiple functions, such as clearing heat and purging fire, downbearing counterflow and freeing discharge, drying dampness, and fortifying yin [1]. Modern pharmacological studies demonstrate that chemical compounds, such as peptides, alkaloids, and flavonoids, form the shared material basis underlying both the bitter taste and pharmacological effects of these substances [2]. With the discovery and in-depth investigation of bitter taste receptors (TAS2Rs), the pharmacology research on these receptors has provided a novel molecular perspective for understanding the mechanisms of bitter Chinese herbal medicine (BCHM).

TAS2Rs, which belong to the G protein-coupled receptor (GPCR) family, comprise 25 members in humans. Localized within taste buds, TAS2Rs serve as the primary receptors mediating bitter taste perception. They mediate this perception in taste cells by activating the Gβ3γ13-PLCβ2-IP3R3 signaling cascade, which induces intracellular Ca^2+^ release [3]. Recent studies have shown that extra-oral tissues, including the respiratory, digestive, and reproductive systems, express TAS2Rs at high levels, where they exert critical regulatory roles in physiological processes such as immunity and metabolism [4,5,6]. For example, TAS2R10, TAS2R14, and TAS2R38 are present in the gastrointestinal tract and modulate gastric acid, bile, and gut hormone secretion, and thus regulate appetite, digestive function, blood lipid metabolism, and even intestinal inflammation [7,8,9,10,11].

Numerous compounds derived from TCM have been identified as agonists of TAS2Rs, such as piceid and liensinine that activate TAS2R10, nobiletin and baicalin that activate TAS2R14, berberine that activates TAS2R38, andrographolide, artemisinin, and strychnine that activate TAS2R46 [12,13]. These bioactive compounds commonly exhibit pharmacological activities, such as antibacterial, anti-inflammatory, and antiviral effects [14,15,16], demonstrating a functional convergence with the modern pharmacological interpretation of BCHM. Although TAS2Rs play a crucial role in physiological processes and the pathogenesis of related diseases, mechanistic insights into their activation have long been limited by the lack of structural data. In recent years, breakthroughs in cryo-electron microscopy (cryo-EM) have facilitated the determination of high-resolution structures of several TAS2Rs bound by bitter compounds, including TAS2R14, TAS2R16, and TAS2R46 [17,18,19,20]. A systematic elucidation of the signal transduction pathways of bitter taste receptors and bitter compounds, from both functional and structural perspectives, has become essential for understanding their physiological and pathological roles as emerging therapeutic targets.

The extraoral function of TAS2Rs opens up a new frontier for understanding how bitter herbal components exert systemic pharmacological effects. Here, we systematically review the distribution and primary physiological functions of TAS2Rs, the binding mode of bitter compounds from BCHMs, as well as the associations between single-nucleotide polymorphisms (SNPs) and the pathogenesis of diseases, such as inflammatory and cancer. This review elucidates the functional relationship between the bitter taste properties of BCHMs and TAS2R pharmacology, integrating classical TCM theoretical interpretations of bitterness with contemporary mechanistic insights into TAS2R activation. It further analyzes current challenges and future opportunities in developing TAS2R-targeted therapeutics from BCHMs.

## 2. Methods

**Figure 1:**
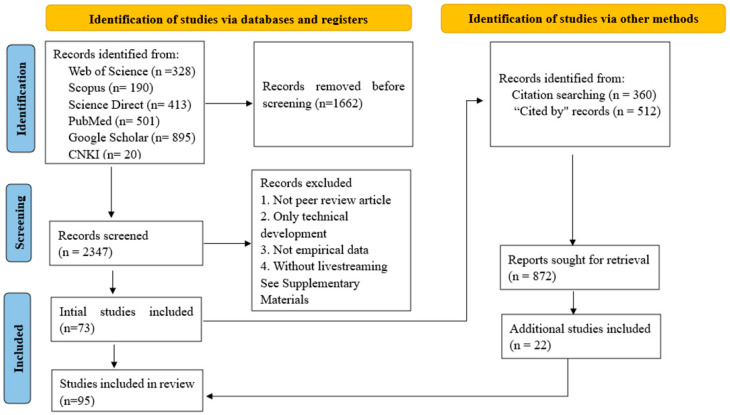
PRISMA flow diagram of the systematic review search procedure.

To ensure the transparency and methodological rigour expected of systematic literature reviews in TAS2Rs and BCHM research, this study followed the PRISMA 2020 guidelines [21]. This review protocol has not been formally registered (no PROSPERO/OSF registration). The PRISMA 2020 flow diagram is presented in Figure 1, and the completed PRISMA 2020 checklist is provided in the Supplementary Materials, as follows in Table S1. The number of articles identified, screened, and included is shown in Figure 1. We used a two-stage search process, including database searches and citation searches. Unlike prior works, this review combs through the diversity of bitter compounds from TCM and features of TAS2Rs, including their tissue distribution, physiological functions, structural characteristics, signal transduction mechanisms, and single-nucleotide polymorphisms.

### 2.1. Literature Search and Study Selection

The literature search was conducted using six databases to ensure both cross-field comprehensiveness and disciplinary depth. Specifically, Scopus and Web of Science served as the foundational databases due to their extensive indexing in communication research and social science. To obtain an interdisciplinary perspective, the ScienceDirect database was included. At the same time, to maintain the rigor of the method, grey literature that has not undergone peer review was excluded. Furthermore, the literature was retrieved from PubMed, Google Scholar, and China National Knowledge Infrastructure (CNKI) databases for cross-validation and forward/backward citation tracing. To ensure comprehensive coverage while maintaining thematic relevance, a Boolean search strategy was adopted. Studies related to TAS2Rs and closely associated constructs were identified using the following query: (“Bitter taste receptors*” OR “TAS2Rs*” OR “TAS2R*” OR “Bitter taste*” OR “Bitter Chinese herbal medicine*”). This search was intentionally broad to capture the full scope of research on TAS2Rs. This syntax was adapted as needed for each database. The initial search was completed on 5 July 2025, and updated on 6 December 2025. Given that this topic has only emerged recently, there is no publication date restriction, which allows for the inclusion of foundational literature while giving priority to empirical studies published within the past five years. In addition, the authors independently screened the titles and abstracts of all content, and in necessary cases, the full texts as well, to determine whether they met the inclusion criteria. Any differences that emerged were resolved through discussion to reach a consensus.

### 2.2. Eligibility and Filtering Criteria

To ensure relevance and methodological rigor for this review, the following filters were applied: (1) language: published in English; (2) publication type: only peer-reviewed journal articles were retained; (3) topical relevance: review had to explicitly focus on TAS2Rs and BCHM; (4) empirical scope: only studies reporting original data of cryo-EM structures for TAS2R14, TAS2R16, and TAS2R46 were obtained from the PDB (https://www.rcsb.org/, 9 October 2025) were included. Studies were excluded if TAS2Rs and BCHM were not closely related, focused solely on technological development, or lacked empirical data. This process adheres to the PRISMA 2020 guidelines and ensures consistency with the research scope of TAS2Rs.

This eligibility framework ensures that the focus remains consistent with the scope of research on TAS2R and BCHM. Forty articles were included from the database search. Then, we identified more records that met the inclusion criteria by reviewing the reference lists of the included literature and conducting citation searches using the “Cited by” option in Google Scholar. For studies with more than 360 citing references on Google Scholar, we searched for “TAS2Rs” in these citing references and reviewed the top 360 results sorted by relevance.

## 3. Results

### 3.1. Natural Ligands of TAS2Rs Derived from BCHM

Bitter compounds serve dual roles as taste stimuli and pharmacologically active agents with therapeutic potential. Their actions are primarily mediated by TAS2Rs, which are directly linked to the characteristic bitterness of BCHM. When bitter compounds bind to TAS2Rs on taste buds, signal transduction can be triggered that is transmitted to the cerebral cortex through neural pathways, leading to the perception of bitterness. Zhang et al. screened for bitter agonists derived from Chinese herbal medicines and identified 2173 compounds capable of activating TAS2Rs across 206 medicinal herbs, with 71.84% of these compounds originating from species characterized by bitter taste [22]. Another screening study revealed that over 70% of tested bitter extracts from BCHM could act as TAS2R agonists, specifically among 26 bitter compounds evaluated [13].

**Figure 2:**
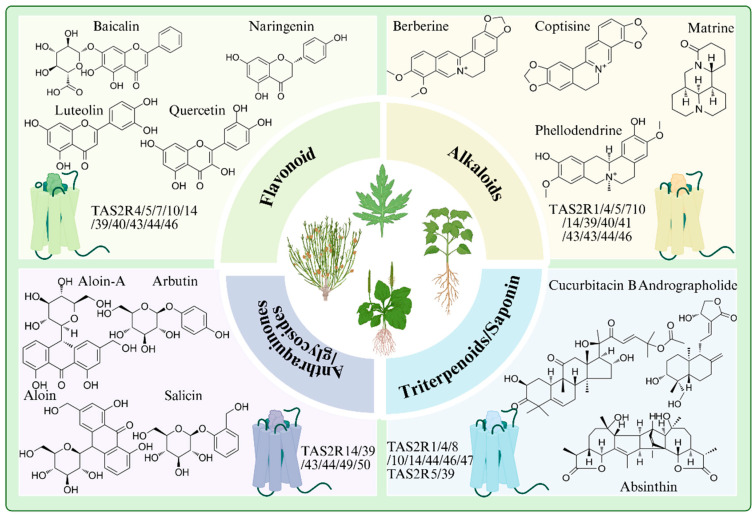
Representative natural ligands of bitter taste receptors (TAS2Rs) derived from Chinese herbal medicines.

TAS2Rs possess the capability to recognize thousands of bitter-tasting molecules, spanning a vast chemical space that encompasses amides, peptides, alkaloids, terpenoids, flavonoids, and other compounds [12]. Under pathological conditions, a subset of TAS2R agonists can exert therapeutic effects by modulating the expression of disease-associated downstream factors through TAS2R activation [23]. Representative natural ligands of TAS2Rs derived from Chinese herbal medicines are shown in Figure 2. The specific binding mechanisms and pharmacological effects of bitter compounds from BCHM, such as coptisine, berberine, phellodendrine, and baicalin [24,25,26], remain to be fully elucidated. The most extensive studies on TAS2R interactions with BCHM agonists focus on berberine [7,27], as an active compound derived from *Coptis chinensis*. Berberine has been shown to activate TAS2R38, which in turn promotes the release of glucagon-like peptide-1 (GLP-1) and contributes to glucose homeostasis regulation [28]. This pathway highlights its promising therapeutic role in type 2 diabetes management. Furthermore, pharmacological investigations on limonin, a bioactive constituent found in *Chenpi*, *Fructus aurantii*, or *Melia toosendan,* and various other BCHM [29,30] have also revealed relatively well-defined molecular mechanisms. Activation of TAS2R14 by limonin and limonin-like compounds induces airway smooth muscle relaxation and bronchodilation [31], demonstrating a potential pathway through which these agents exert anti-asthmatic effects. TAS2R50 mediates the anti-inflammatory effects of resveratrol by inhibiting interleukin-6 release in HGF-1 cells, thereby demonstrating its possible anti-inflammatory efficacy [32].

These studies highlight the potential of TAS2Rs as promising therapeutic targets for various diseases, yet significant challenges remain. Firstly, although a growing number of natural TAS2R agonists have been identified, many induce receptor activation without exhibiting proven disease-modifying effects. Secondly, the widespread tissue distribution but low expression level of TAS2Rs, the lack of high-potency ligands, and the chemical structural diversity of bitter compounds, all impede targeted pharmacological modulation for clinical translation. Further research into the pharmacological mechanisms of bitter compounds acting on TAS2Rs is therefore essential.

### 3.2. The Expression and Physiological Functions of TAS2Rs

**Figure 3:**
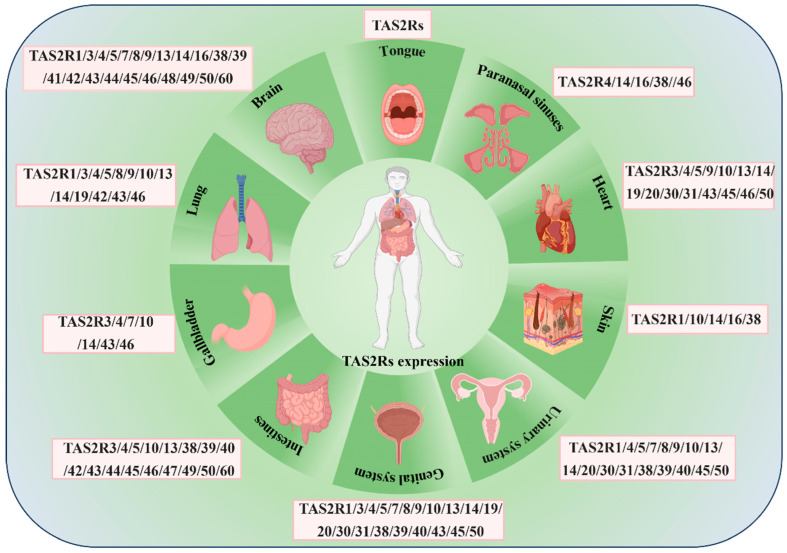
Expression and distribution of TAS2Rs in human body systems.

“Bitterness” is a crucial property of TCM, traditionally regarded as a signal of toxin avoidance. Modern research has revealed that TAS2Rs are not only expressed in the oral cavity but are also widely distributed in tissues such as the respiratory tract and intestines. The “bitterness” of TCM may be key to their therapeutic effects, acting activating through TAS2Rs to modulate physiological functions. Consequently, the functional role of TAS2Rs has been expanded from simple gustatory receptors to systemic chemical signal detectors. TAS2Rs are predominantly localized to the lingual papillae of gustatory tissues, where they are concentrated in type II taste cells within taste buds [33]. Notably, their expression extends widely to extra-oral tissues, encompassing diverse systems such as the respiratory, digestive, cardiovascular, and immune systems (shown in Figure 3). In the respiratory system, TAS2Rs facilitate airway defense and clearance by regulating ciliary beating and exerting bactericidal activity against pathogenic microorganisms [34,35]. Within the digestive system, beyond their roles in microbiota regulation and immune defense, TAS2Rs influence gastrointestinal function and glucose metabolism by modulating the secretion of gastric acid [36] and hormones [37]. In the cardiovascular system, TAS2Rs not only perform physiological regulatory functions in cardiac and vascular smooth muscle [38] but are also implicated in the hematopoietic microenvironment, such as in the bone marrow [39,40,41]. Furthermore, TAS2Rs also play significant roles in immune regulation [42], reproductive protection [4,43], skin barrier maintenance [44], and neuroprotection [45]. Their role in suppressing inflammatory responses across multiple systems highlights the extensive and diverse physiological regulatory capacities. The expression, distribution, and associated physiological functions of TAS2Rs across human tissues are summarized in Table S2.

#### 3.2.1. Respiratory System

Within the respiratory system, TAS2Rs exhibit extensive expression in various tissues, including the sinonasal cavity [46], airway epithelial cilia [47], and airway smooth muscle [34]. Serving as the respiratory tract’s primary chemical defense, they facilitate defense and clearance via sensing inhaled toxins. In the upper airways, solitary chemosensory cells (SCCs) in the human sinonasal epithelium express TAS2Rs that detect airborne irritants and microbial metabolites, thereby initiating local innate immune responses as chemosensory sentinels of the airway [46]. Furthermore, ciliated cells in the sinus mucosa express TAS2R4, TAS2R16, and TAS2R38, which exert antimicrobial effects through the canonical bitter taste signaling pathway by mediating the production of nitric oxide (NO) [48]. In the lower airways, TAS2Rs in airway epithelial cilia modulate ciliary beating in response to bitter stimuli, facilitating the defense against and clearance of harmful substances [47].

#### 3.2.2. Digestive System

In the digestive system, TAS2Rs exhibit a broad distribution across gastrointestinal tissues and cell types, spanning the stomach to the intestines and encompassing enteroendocrine cells [49,50,51], goblet cells, paneth cells [5], and tuft cells [7,52]. These distributions may relate to the digestive regulation, immune defense, and metabolic homeostasis through the detection of luminal contents. In the stomach, Liszt et al. demonstrated the expression of TAS2R10 and TAS2R43 in parietal cells and chief cells of the human gastric mucosa, which is closely associated with the regulation of gastric acid secretion [10]. Gastrointestinal smooth muscle cells are also key sites of TAS2R expression, such as TAS2R38 and TAS2R46. Bitter compounds activate these receptors, leading to internal storage in the IP3 pathway. This signaling pathway may induce a rapid membrane depolarization and increased cytosolic calcium levels, resulting in an accelerated cell contraction, as well as regulating gastric motility and emptying [50].

As a key site for nutrient absorption and immune defense, small intestine also exhibits broad expression of various TAS2Rs. In jejunal crypts, TAS2R43 in goblet cells induces CLCA1/MUC2 release for mucus remodeling and microbiota regulation, while TAS2R10 mediates antimicrobial peptide secretion to inhibit bacterial growth and enhance innate immunity [5]. TAS2Rs expressed at gastrointestinal interfaces can also establish indirect regulatory networks. They not only regulate the Intestinal microbiota homeostasis and metabolites, but also promote gastrointestinal hormone secretion, such as GLP-1, ghrelin, and cholecystokinin, constituting a “gut–vascular axis” [53]. Kim et al. demonstrated high expression of TAS2R14 in the small intestine, which can recognize endogenous bitter compounds and enhance signal transduction through allosteric synergy with bitter compounds to potentially regulate gastrointestinal metabolism [19].

#### 3.2.3. Cardiovascular System

Within the cardiovascular system, TAS2Rs act as endogenous metabolic sensors and vascular tone regulators via multiple signaling pathways. Human cardiac tissue expresses multiple TAS2Rs, including subtypes TAS2R10, 14, 30, 31, 46, and 50 [54]. Foster et al. firstly identified TAS2R transcripts/proteins in failing human hearts and normal rodent myocardium, noting high expression of over half of TAS2Rs in the human heart, with TAS2R14 being particularly abundant. TAS2R expression may be developmentally and nutritionally regulated, with specific genes upregulated under starvation, suggesting involvement in cardiac metabolic stress responses [39]. Recent research further indicates that TAS2R14, abundant in the human heart, couples to Gi protein to inhibit adenylate cyclase (AC) activity and reduce cyclic adenosine monophosphate (cAMP) levels [54]. This mechanism might play a role in reducing heart rate and myocardial contractility, thus regulating cardiac metabolism. TAS2Rs 10, 38, 40, and 42 distributed in porcine coronary arteries induce concentration-dependent vasorelaxation, suggesting potential therapeutic targets for the management of coronary artery diseases [55].

#### 3.2.4. Immune System

In the immune system, TAS2Rs play significant roles in immune responses and inflammatory regulation. According to the study by Malki et al., the expression of the full repertoire of 25 human TAS2Rs occurs in various leukocyte subsets, with TAS2R31 and TAS2R43 showing the most prominent expression. Activation of these two receptors by saccharin modulates neutrophil chemotaxis and migration, thereby contributing to immune responses [56]. In neutrophils, TAS2R38 serves as a receptor for the quorum-sensing molecule AHL-12, mediating neutrophil activation, enhancing early pathogen clearance, and inhibiting biofilm formation [57]. In lymphocytes, activation of TAS2R38 by its natural agonist goitrin induces the PLCβ2-IP3 signaling pathway, selectively suppresses the pro-inflammatory cytokine TNF-α, and thereby exerts anti-inflammatory effects [58]. Grassin-Delyle et al. detected the expression of 16 TAS2Rs in lung macrophages isolated from postoperative cancer patients. Among these, TAS2R3, 4, 5, 9, 10, 14, 30, 39, and 40 were primarily associated with anti-inflammatory responses, with TAS2R7 and TAS2R38 exhibiting particularly prominent expression and functional significance [59]. Talmon et al. found that the TAS2R46 expression levels were significantly higher in monocytes than in macrophages. Moreover, TAS2R46 protects monocytes/macrophages from oxidative stress damage, regulates cell differentiation, and reduces inflammatory responses [60].

#### 3.2.5. Urogenital System

The urogenital system, a prevalent site of bacterial infection, exhibits widespread TAS2R expression. These receptors play a critical role in mediating anti-infective and anti-inflammatory responses by detecting quorum-sensing molecules secreted by pathogenic microorganisms [43]. Deckmann et al. demonstrated that urethral cholinergic brush cells in rodents express TAS2Rs. When stimulated by harmful substances, these cells release acetylcholine to activate sensory nerves, which reflexively contract the detrusor muscle. This constitutes a protective bladder reflex that expels irritants and helps prevent infection [61]. Conversely, TAS2Rs are also directly present in the detrusor muscle, where their activation induces smooth muscle relaxation [62].

The expression and functional impact of TAS2Rs exhibit distinct differences between the male and female reproductive systems. In the male system, TAS2Rs present in testicular tissue and sperm play a significant role in regulating spermatogenesis and sperm maturation. They are also implicated in mediating the acrosome reaction, potentially via the PDE-cAMP and PLCβ2-IP3 signaling pathways [63]. The female reproductive system constitutes a more complex organ network. The broad expression of TAS2Rs is seen in the ovary [64,65], uterus, and placenta [4]. They participate in processes including lipid metabolism, which affects hormone synthesis, and may regulate oocyte development and follicular cell function. Additionally, they appear to contribute to embryonic protection [4,43].

#### 3.2.6. Nervous System

The expression of TAS2Rs extends beyond peripheral tissues to the nervous system, where they exert neuroregulatory and barrier-protective functions. Singh et al. detected mRNA expression of the human orthologous receptors TAS2R4, TAS2R10, and TAS2R38 in multiple brain regions of rats [66]. Beyond neuronal cells, TAS2Rs can also be observed in the human blood-cerebrospinal fluid-barrier. The expression of TAS2R4, 5, 14, and 39 was confirmed in the choroid plexus epithelial cells. By sensing bitter compounds in the bloodstream or cerebrospinal fluid, these receptors may regulate barrier integrity, modulate inflammatory responses, and influence ABC transporter-mediated substance transport across the barrier, thereby contributing to central nervous system homeostasis [67].

### 3.3. The Structural Characteristics and Signaling Transduction Regulatory Mechanisms of TAS2Rs

Elucidating the intracellular signaling pathways triggered by bitter components acting on TAS2Rs not only opens up the “black box” between “bitterness” and “TCM efficacy,” but also unveils the potential scientific connotation of TCM at molecular level. The significance is primarily manifested in the following aspects: (1) Revealing the direct pharmacological mechanisms of bitter TCM. This approach allows confirmation that bitter components exert their effects precisely by activating specific TAS2Rs. For instance, berberine activates TAS2R38 to produce a hypoglycemic effect. (2) Promoting innovative drug development based on TAS2Rs. These receptors can serve as targets for screening novel therapeutic agents. For example, developing specific agonists for TAS2R14, which is highly expressed in the airways, could lead to new bronchodilators for treating asthma [12]. Furthermore, once these signaling pathways are clarified, bitter active ingredients from TCM can be utilized as lead compounds, such as coptisine, berberine, phellodendrine, and baicalin [24,25,26]. Through structural modification, this strategy can yield more potent and specific TAS2R-targeting drugs. Therefore, elucidating the signaling pathways of bitter compounds acting on TAS2Rs achieves a leap from gustatory perception to cellular response.

#### 3.3.1. Signaling Transduction Regulatory Mechanism

**Figure 4:**
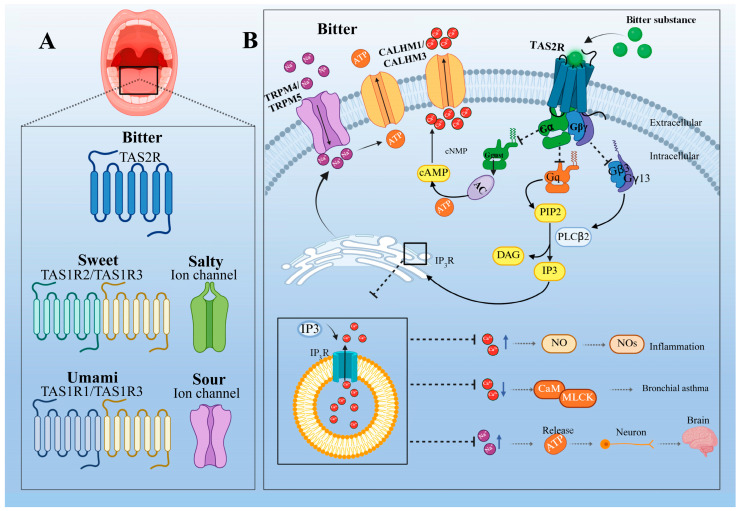
Schematic representation of the TAS2R signaling pathway. (**A**) The taste buds on the human tongue are primarily responsible for perceiving the five basic tastes. (**B**) The main signaling mechanism of bitter taste receptors perceiving bitter substances. Note: TAS2Rs, bitter taste receptors; AC, adenylate cyclase; PDE, phosphodiesterase; cAMP, cyclic adenosine monophosphate; ATP, adenosine triphosphate; PIP2, phosphatidylinositol 4,5-bisphosphate; PLCβ2, phospholipase Cβ; DAG, diacylglycerol; IP3, inositol trisphosphate; IP3R, inositol trisphosphate receptor; TRPM4/TRPM5, transient receptor potential cation channel subfamily M member 4/5; CALHM1/CALHM3, calcium homeostasis modulator 1/3.

In gustatory tissues, the heterotrimeric G protein complex comprises Ggust, Gβ3, and Gγ13 subunits. Agonist binding induces conformational changes in the heterotrimeric G protein, leading to its dissociation into a Ggust subunit and a Gβγ dimer, subsequently initiating two classical signaling pathways, PDE-cAMP and PLCβ2-IP3, with the Gβ3γ13-mediated PLCβ2-IP3 pathway representing the primary signaling route. Specifically, Ggust can activate phosphodiesterase (PDE) in taste tissues, promoting the hydrolysis of cAMP and consequently reducing intracellular cAMP levels [23]. The underlying mechanism is not fully elucidated but may involve the disinhibition of cyclic nucleotide-inhibitory channels (cNMP), ultimately leading to an increase in intracellular Ca^2+^ concentration [68,69]. Meanwhile, the Gβ3γ13 dimer activates PLCβ2, catalyzing the hydrolysis of phosphatidylinositol 4,5-bisphosphate (PIP_2_) to generate inositol trisphosphate (IP_3_) and diacylglycerol (DAG). IP_3_ then binds to type III IP_3_ receptors (IP_3_RIII) on the endoplasmic reticulum surface, triggering substantial Ca^2+^ release from the endoplasmic reticulum [70]. The increased Ca^2+^ further activates TRPM4/TRPM5 channels on the plasma membrane, leading to Na^+^ influx and membrane depolarization [71]. This depolarization activates CALHM1/CALHM3 ion channels, resulting in ATP release [70]. ATP acts as a neurotransmitter, and the signal is ultimately transmitted to the cerebral cortex via synaptic transmission, completing gustatory perception (Figure 4).

G protein dissociation upon TAS2R activation initiates signaling via either the classical pathways or alternative mechanisms. In bone tissue, TAS2R38 and TAS2R46 activate the PLCβ-IP_3_ pathway to induce calcium signaling, which may regulate osteocyte proliferation, differentiation, and metabolism [72]. In airway smooth muscle during inflammation, elevated histamine secretion raises intracellular Ca^2+^. Here, artabsin-induced activation of TAS2R46 may upregulate the mitochondrial calcium uniporter (MCU) via the cAMP-EPAC pathway. This enhances mitochondrial Ca^2+^ uptake, thereby reducing cytosolic Ca^2+^ levels [73]. In skeletal muscle cells, TAS2R46 mediates calcium signaling through the cAMP-EPAC-MCU axis, attenuating acetylcholine-induced rapid Ca^2+^ rises and subsequent muscle fiber contraction, thus preventing hypercontraction or spasms [74]. Collectively, these non-gustatory signaling pathways extend the functional repertoire of TAS2Rs, enabling them to contribute to critical pathophysiological processes such as the regulation of cell proliferation and the mitigation of inflammatory responses.

#### 3.3.2. Structure Features and Ligand Binding Modes of TAS2Rs

**Figure 5:**
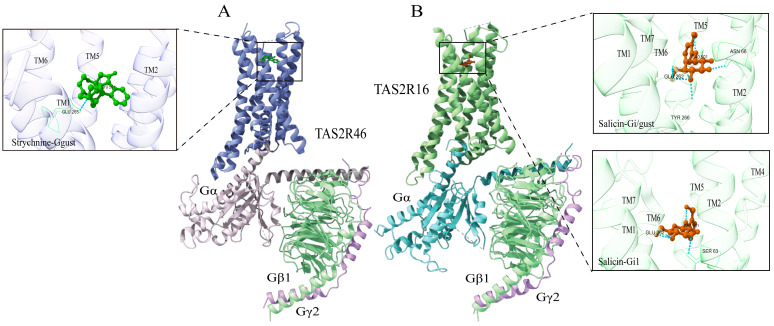
Structural features of TAS2R46 (**A**) and TAS2R16 (**B**), and binding modes of strychnine (colored in green) and salicin (colored in brown) at TAS2R46 and TAS2R16, respectively. Notes: strychnine-TAS2R46-Ggust (PDB: 7XP6); Salicin-TAS2R16-Gi1/gust (PDB: 9KPE); Salicin-TAS2R16-Gi1 (PDB: 9KPF).

Structurally, TAS2Rs share similarity with class A GPCRs but lack their conserved activation motifs, such as DRY and NPxxY, resulting in distinct activation mechanisms [75]. In recent years, significant advancements in cryo-EM techniques have facilitated the determination of activated-state structures for three members of the TAS2R family, including TAS2R46 [18], TAS2R16 [20], and TAS2R14 [17,19]. TAS2R46, as a broad-spectrum bitter taste receptor [76], has attracted sustained interest due to its unique molecular architecture, wide ligand recognition profile, and complex activation mechanism. The high-resolution cryo-electron microscopy structure of TAS2R46 in complex with strychnine provides a critical structural foundation for deciphering the activation mechanism of class T GPCR [18]. The transmembrane helix arrangement in TAS2R46 displays marked specificity: the extracellular end of TM5 is closely positioned relative to TM3, whereas the extracellular end of TM4 is distal to TM3 and TM5, thereby forming a funnel-shaped ligand-binding pocket on the extracellular side that involves TM3, TM4, and TM5. This structural configuration confers a distinct spatial architecture conducive to the binding of bitter molecules (Figure 5A). In the inactive state, the side chain of Y241^6.48^ points outward. It acts as a conformational switch, undergoing an approximately 90° rotation upon activation to form a stable hydrogen bond with N92^3.36^, thereby triggering receptor activation [77]. Furthermore, the study revealed that TAS2R46 can pre-couple with Gs/gust, a non-canonical activation mode that may facilitate rapid signal transduction upon ligand binding [18]. Comparative analysis of the activated and pre-activated state structures elucidated the signal transduction mechanism: Strychnine binds within the orthosteric, funnel-shaped pocket of TAS2R46. The conserved residue W88^3.32^ acts as a base, forming a π-π interaction with the benzene ring of strychnine, while a hydrogen bond with E265^7.39^ stabilizes the binding, enabling rapid recognition of diverse bitter molecules [18].

In a recent study, the cryo-EM structure of the TAS2R16 complex with salicin and G proteins (Ggust, Gi1, Gi2) was shown, showing the molecular mechanism underlying its specific recognition of salicin, as shown in Figure 5B. The salicin ring forms a π-π interaction with the highly conserved tryptophan residue W85^3.32^ and forms a critical hydrogen bond with E262^7.39^, stabilizing the bitter molecule binding. This study also identified conserved class T motifs-H^7.49^S^7.50^TSL^7.53^ and F^3.49^Y^3.50^C^3.51^ that stabilize the active state conformation. It further provided the first detailed characterization of the receptor’s interaction mode with Ggust and Gi proteins [20], offering new insights into the diversity of TAS2R signaling mechanisms.

**Figure 6:**
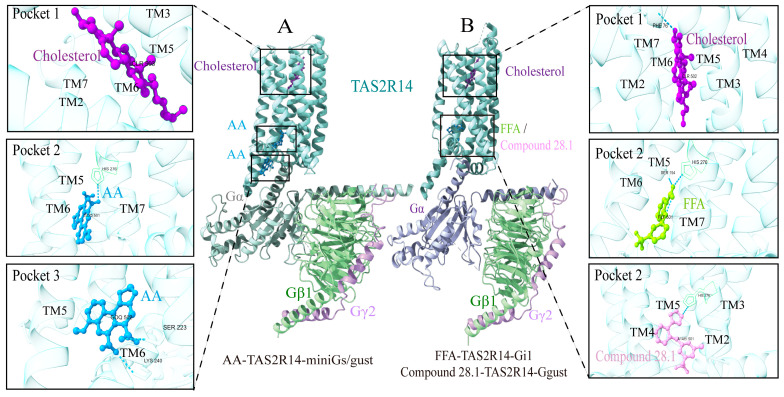
Cryo-EM structures of TAS2R14-G protein complexes. (**A**): Structural features of TAS2R14 and binding modes of cholesterol (colored in purple) in pocket1 and aristolochic acid (AA, colored in blue) in pocket 2 and 3, respectively; (**B**): Structural features of TAS2R14 and binding modes of cholesterol (colored in purple), flufenamic acid (FFA, colored in kelly), or compound 28.1 (colored in pink), respectively. Notes: The accession code of AA-TAS2R14-miniGs/gust (PDB: 8XQL) with (**A**); The accession code for FFA-TAS2R14-Gi1 (PDB: 8XQS) and compound 28.1-TAS2R14-Ggust (PDB: 8YKY) in pocket 2 with (**B**), respectively.

Recently, multiple studies have reported three-dimensional structures of TAS2R14 bound to various bitter ligands. Compared to TAS2R46 and TAS2R16, TAS2R14 exhibits a more complex ligand binding mode. Structure analysis reveals that TAS2R14 possesses several binding pockets. Structural analysis of the receptor bound to aristolochic acid with Gi or Gs/gust revealed three distinct ligand-binding pockets with TAS2R14, as shown in Figure 6A [17]. Pocket 1 is formed by the extracellular segments of TM2, TM3, TM5, and TM7, resembling the canonical orthosteric binding site of most class A GPCRs. Cholesterol binds to this pocket in a concentration-dependent manner to activate TAS2R14, with the conserved residue W89^3.32^ playing a critical role, analogous to W88^3.32^ in strychnine-bound TAS2R46. Pocket 2, as an allosteric site [17], is located on the intracellular side of TM7 and formed by TM3, TM5-TM7, and the C-terminal end of the Gα subunit α5-helix. The conserved residues Y107^3.50^ and H276^7.49^ stabilize the binding of aristolochic acid within this pocket. Pocket 3 is formed by the intracellular sides of TM5 and TM6, and the α5-helix, potentially resulting in a wider cytoplasmic opening between TM5 and TM6 compared to TAS2R46. This pocket may serve as a hydrophobic channel during receptor activation. When cholesterol binds to the orthosteric site, conformational changes are potentially relayed through this channel to the intracellular allosteric site, enabling bitter agonists to stabilize the G protein-binding interface and achieve synergistic activation. Furthermore, TAS2R14 can couple to both Ggust and Gi proteins, with the sequence and conformational diversity of ICL2 significantly influencing G protein selectivity [17].

To elucidate the activation mechanism of TAS2R14, we compared TAS2R14 complex structures by combining different agonists. It was also reported that the cryo-EM structure of the TAS2R14 complex with flufenamic acid or compound 28.1 through different G proteins (Gαgust and Gi1) in pocket 2 (Figure 6B). A conserved structural feature is the occupancy of pocket 1 by cholesterol. While the exact role of bile acids or cholesterol in TAS2R14 remains unclear, it is speculated that this receptor may be involved in their metabolism or in the pathophysiology of associated metabolic disorders, such as obesity and diabetes. This research provides us with the following insights: (1) TAS2Rs are viable targets for multi-target drugs regulating diverse processes like cholesterol homeostasis, airway contraction, and gastrointestinal reactions. (2) Intracellular pockets can be exploited to design selective G protein pathway modulators, potentially minimizing side effects. (3) The NSAID-like structure of compound 28.1 provides a basis for developing novel bitter-tasting drugs or anti-inflammatory substances.

In short, a deep understanding of the ligand recognition patterns, receptor activation mechanisms of TAS2Rs, and the molecular mechanisms by which function enhances our comprehension of the structure and function of bitter taste receptors. It also provides a novel perspective for the design of drug candidates targeting TAS2Rs.

### 3.4. SNPs and Pathological Mechanisms of TAS2Rs

The SNPs of the TAS2Rs genes serve as a key genetic bridge linking bitter components to disease risk. They explain why individuals have different responses to the same bitter components and why there are differences in susceptibility to certain diseases. SNPs, a major form of genetic variation, are thought to modulate dietary behaviors, and these changes may contribute to the development of chronic diseases. A study involving 363 pregnant women found that individuals carrying the CC genotype at the rs3741845 (Ala187Val) locus of the TAS2R9 gene had a significantly higher risk of developing Gestational Diabetes Mellitus (GDM) (*p* = 0.0087). Furthermore, significant differences in dietary patterns were observed in GDM patients, characterized by more frequent consumption of meat, dairy products, and sugar-sweetened beverages. This association suggests that genetic variations in TAS2R9 may influence dietary choices by altering taste preferences, thereby potentially increasing the risk of GDM [78].

**Figure 7:**
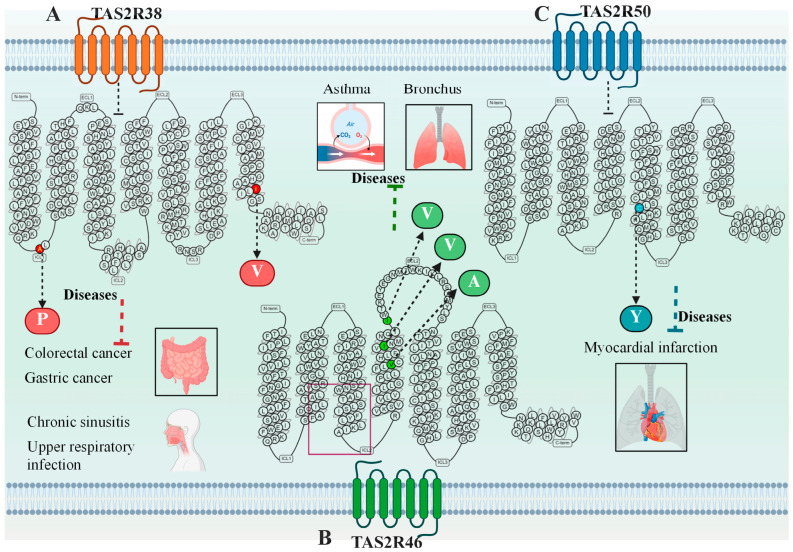
Single-nucleotide polymorphisms of the TAS2Rs and the related diseases.

However, genetic variations can also directly influence disease pathogenesis through non-gustatory pathways. Existing evidence indicates that specific TAS2R SNPs are linked to the development of disease conditions, such as inflammation and cancer. For instance, three common SNPs in TAS2R38, including rs713598 (Ala49Pro), rs1726866 (Val262Ala), and rs10246939 (Ile296Val), which are associated with interindividual differences in chemosensory response and innate immune defense, may contribute significantly to susceptibility to inflammatory diseases and cancer [79,80,81,82]. Genetic variants in different TAS2Rs’ genes often exhibit a predisposition towards specific types of diseases, such as variants of TAS2R46 affect pathophysiological functions of the respiratory tract, variants of TAS2R50 are closely linked to cardiovascular diseases, etc. Primary TAS2Rs’ SNPs and their disease associations are summarized in Figure 7 and Table S3.

#### 3.4.1. SNPs of TAS2R38

In research on SNPs of TAS2Rs, the association between TAS2R38 and diseases is particularly prominent, significantly influencing susceptibility to respiratory infections and gastrointestinal cancers. In the respiratory system, three common SNPs in TAS2R38 influence susceptibility to respiratory infections by modulating key airway defense mechanisms, including ciliary beating and innate immune responses. The AVI allele is notably associated with an increased risk of respiratory system infections [83]. Activation of TAS2R38 by N-acyl-homoserine lactones (AHLs) upregulates Ca^2+^ and nitric oxide (NO), which mediate enhanced ciliary clearance and antibacterial effects, thus protecting against upper respiratory tract infections. In contrast, the AVI allele variant is associated with a significant reduction in NO output and weakened ciliary motility. This pathway deficiency underlies the increased predisposition to Gram-negative sinus infections observed in these patients [83]. Another study similarly found that the sinus mucosa of patients with Chronic Rhinosinusitis (CRS) highly expresses TAS2R38, and that the AVI haplotype acts as an independent risk factor for CRS development [84]. A pivotal study by Parsa et al. during the pandemic established a pioneering link between TAS2R38 SNPs and COVID-19 mortality, suggesting that the PAV allele may enhance antiviral defense in the respiratory tract, with the rs10246939 (Ile296Val) G allele potentially playing a key role [85].

In the digestive system, genetic variations in TAS2R38 may influence susceptibility to gastrointestinal cancers by modulating the clearance of gastrointestinal toxins, although the precise pathological mechanisms remain unclear. Multiple variant alleles might synergistically affect disease development. In particular, reveal that the effects of TAS2R38 SNPs are not entirely consistent across different populations. Choi et al. found that while the AVI/AVI diplotype showed no significant association with gastric cancer (GC) risk, the PAV/AVI heterozygous genotype was associated with an increased risk [81]. Conversely, within another Korean study, the AVI/AVI diplotype was associated with a significantly reduced risk of colorectal cancer, and this protective effect was enhanced in the presence of the carbonic anhydrase 6 rs2274333 (Ser90Gly) G allele [86]. This suggests that the cancer risk effect of the AVI/AVI diplotype may exhibit population dependency.

#### 3.4.2. SNPs of TAS2R46

The expression and signaling activation of TAS2R46 in the bronchi also influence the pathophysiological functions of the respiratory tract. Investigations by Lecchi et al. into the role of TAS2R46 in histamine-induced downstream signaling demonstrated that three known single-nucleotide polymorphisms (SNPs) in the fourth transmembrane domain, including rs200936852 (Val141Ala), rs72477411 (Ile147Val), and rs72477410 (Ile153Val), while not affecting artabsin binding, lead to a significant attenuation or even abolition of mitochondrial calcium uptake [87]. Thus, the rescue by an EPAC agonist implicates the cAMP-EPAC pathway as being compromised by these variants, leading to aberrant calcium signaling, thereby predisposing individuals to asthma or chronic respiratory diseases [73,87,88].

#### 3.4.3. SNPs of TAS2R50

TAS2R50 is present within the heart, and its genetic variants are closely linked to cardiovascular diseases. Shiffman et al. investigated 74 candidate gene variants and identified four (in genes KIF6, VAMP8, LPA, and TAS2R50) that were significantly associated with myocardial infarction risk in an elderly Caucasian population. Specifically, the rs1376251 (synonymous mutation) of TAS2R50 can increase disease risk by 13-14% [89]. Compared to wild-type, Bloxham et al. revealed that agonist stimulation of the TAS2R50 Tyr203 variant results in a markedly attenuated maximum response (Emax), reduced by about 50%. This confirms that TAS2R50 SNPs cause functional signaling differences relevant to cardiovascular disease modulation [54]. Beyond TAS2R50, common genetic variants in other highly expressed cardiac receptors, such as TAS2R14, TAS2R30, and TAS2R46, also exhibit varying degrees of functional impairment, including diminished response efficacy or loss of signaling function. These deficits may contribute to the molecular basis of interindividual differences in cardiovascular pathophysiology [54].

#### 3.4.4. SNPs of Other TAS2Rs

TAS2R16 has been reported to participate in the modulation of digestive tract and periodontal diseases. Barontini et al. found that the C allele of rs1525489 is significantly associated with an increased risk of rectal cancer [90]. Carriers of the CC haplotype, constituted by rs2270009 (synonymous mutation) of TAS2R3 and rs2234001 (Val96Leu) of TAS2R4, exhibited a significantly reduced risk of developing PTC. These individuals also had significantly lower serum levels of total triiodothyronine, suggesting that TAS2R3/4 gene variants may alter PTC susceptibility by affecting thyroid hormone production or regulation [91]. Furthermore, genetic variations in TAS2R3 and TAS2R14 can affect sperm normal morphology and flagellar movement, indicating a close relationship with the occurrence of male infertility [92].

These investigations provide a novel genetic perspective on interpreting susceptibility differences in digestive, respiratory, and cardiovascular diseases, highlighting the synergistic effects of different receptor subtype polymorphisms and their population-specificity in disease etiology. The findings offer a key foundation for assessing disease risk, identifying early diagnostic biomarkers, and developing personalized interventions through TAS2Rs. In addition, SNPs of the TAS2R gene can lead to changes in the amino acid sequence of the receptor protein, thereby affecting the receptor’s binding ability with bitter substances. This explains why some people find a certain food extremely bitter, while others cannot detect it at all.

## 4. Discussion

Although TAS2Rs constitute a primary pharmacological pathway for bitter compounds, mechanistic insights remain limited due to current insufficient in-depth investigations. At the structural level, the features and dynamic conformational changes of numerous TAS2R subtypes remain to be elucidated, which is crucial for deeply understanding their ligand recognition and activation mechanisms. In signal transduction, the elucidation of novel TAS2R-activated pathways in extra-gustatory tissues and their interactive networks remains incomplete, thereby constraining a systems-level understanding of their mechanisms in pathophysiology. In disease correlation studies, most research remains at the level of establishing associations between TAS2R genetic variations and disease development, while the underlying molecular mechanisms are not fully elucidated, particularly the evident population-specific differential mechanisms.

Although some bitter compounds have been identified as agonists of TAS2Rs, the resulting pharmacological effects and related mechanisms of action are still insufficiently studied. Thus, developing BCHMs targeting TAS2Rs holds promise but faces challenges: (1) BCHMs contain multiple components, making it hard to precisely predict efficacy due to potential activation of various TAS2R subtypes or GPCRs; (2) Systemic TAS2R distribution may cause oral or intestinal receptor activation, leading to side effects like nausea; (3) Most research is limited to in vitro and animal studies, lacking large-scale human trials; (4) The strong bitterness often requires masking in formulations, complicating efforts to balance systemic effects with taste acceptability. Despite these challenges, BCHMs hold promise for TAS2R-targeted therapeutics: (1) Bioinformatics-assisted screening using TAS2R models and TCM databases can identify high-affinity, selective agonists for specific TAS2Rs; (2) Identifying TAS2R targets in classic bitter prescriptions adds modern scientific value; (3) Targeted delivery systems based on TAS2R tissue distribution can be developed. Future studies should adopt human-relevant experimental systems to dissect the roles of TAS2R signaling, both in bitter taste perception and downstream pharmacological effects, and thereby establish a mechanistic, multiscale understanding of how human TAS2R activation contributes to BCHM efficacy.

## 5. Conclusions

Modern pharmacological research increasingly highlights the therapeutic benefits of BCHM. It plays a key role of its active compounds in preventing and treating several diseases. A multitude of active components from BCHM have been recognized as TAS2R agonists with diverse pharmacological effects. The anti-inflammatory and anti-tumor properties of BCHM, particularly in the respiratory and digestive systems, have been extensively studied [93,94]. While numerous studies confirm that bitter herbal extracts exert pharmacological effects via TAS2R activation, investigations into the specific functions and mechanisms of BCHM mediated through TAS2Rs remain limited. This gap is largely owing to the broad receptor distribution, complex signaling pathways, and diverse array of bitter compounds involved. As pivotal members of the GPCR superfamily, TAS2Rs function as regulators of diverse physiological processes through both local actions and systemic signaling. They play critical roles in defense and clearance within the respiratory system, digestive regulation and metabolic maintenance in the gastrointestinal system, endogenous metabolic sensing and vascular tone regulation in the cardiovascular system, and immune response participation and inflammation modulation in the immune system. Studies of SNPs confirmed that TAS2Rs contribute to the pathology of multiple diseases, notably within the digestive, respiratory, and cardiovascular systems. The susceptibility and progression of diseases in these contexts appear to be governed by the combinatorial effect of polymorphic variations across receptor subtypes and their population-specific distributions.

## Data Availability

No new data were created or analyzed in this study. Data sharing is not applicable to this article.

## Supplementary Materials

The following supporting information can be downloaded at: https://www.mdpi.com/article/10.3390/ijms27073073/s1.

## Abbreviations

TAS2Rs	Bitter taste receptors.
GPCRs	G protein-coupled receptors
BCHM	Bitter Chinese herbal medicine
TCMs	traditional Chinese medicines
AC	adenylate cyclase
PTC	phenylthiocarbamide
GLP-1	Glucagon-Like Peptide-1
cAMP	cyclic adenosine monophosphate
cNMP	nucleotide-inhibitory channels
PIP_2_	phosphatidylinositol 4,5-bisphosphate
IP_3_	inositol trisphosphate
DAG	diacylglycerol
IP_3_RIII	type III IP_3_ receptors
SNPs	Single nucleotide polymorphisms
GDM	Gestational Diabetes Mellitus
cryo-EM	cryo-electron microscopy
CRC	colorectal cancer
GC	gastric cancer

## References

[1] Wang H., Wei W.F., Liu J., Zhang S., Zhao Y.L., Yu Z.G. (2024). The characterization of traditional Chinese medicine natures and flavors using network pharmacology integrated strategy. J. Tradit. Complement. Med..

[2] Ahmad R., Dalziel J.E. (2020). G protein-coupled receptors in taste physiology and pharmacology. Front. Pharmacol..

[3] Talmon M., Pollastro F., Fresu L.G. (2022). The Complex Journey of the Calcium Regulation Downstream of TAS2R Activation. Cells.

[4] Lu P., Moore Simas T.A., Delpapa E., ZhuGe R.H. (2024). Bitter taste receptors in the reproductive system: Function and therapeutic implications. J. Cell. Physiol..

[5] Liszt K.I., Wang Q.L., Farhadipour M., Segers A., Thijs T., Nys L., Deleus E., Van der Schueren B., Gerner C., Neuditschko B. (2022). Human intestinal bitter taste receptors regulate innate immune responses and metabolic regulators in obesity. J. Clin. Investig..

[6] Kim D., An S.S., Lam H., Leahy J.W., Liggett S.B. (2020). Identification and Characterization of Novel Bronchodilator Agonists Acting at Human Airway Smooth Muscle Cell TAS2R5. ACS Pharmacol. Transl. Sci..

[7] Sun S.Y., Yang Y.X., Xiong R.Y., Ni Y.Y., Ma X.J., Hou M., Chen L., Xu Z.P., Chen L., Ji M.J. (2022). Oral berberine ameliorates high-fat diet-induced obesity by activating TAS2Rs in tuft and endocrine cells in the gut. Life Sci..

[8] Kiriyama Y., Tokumaru H., Sadamoto H., Nochi H. (2025). Biological Actions of Bile Acids via Cell Surface Receptors. Int. J. Mol. Sci..

[9] Huang T.T., Gu P.P., Zheng T., Gou L.S., Liu Y.W. (2022). Piperine, as a TAS2R14 agonist, stimulates the secretion of glucagon-like peptide-1 in the human enteroendocrine cell line Caco-2. Food. Funct..

[10] Liszt K.I., Ley J.P., Lieder B., Behrens M., Stöger V., Reiner A., Hochkogler C.M., Köck E., Marchiori A., Hans J. (2017). Caffeine induces gastric acid secretion via bitter taste signaling in gastric parietal cells. Proc. Natl. Acad. Sci. USA.

[11] Wang Q., Farhadipour M., Thijs T., Ruilova Sosoranga E., Van der Schueren B., Ceulemans L.J., Deleus E., Lannoo M., Tack J., Depoortere I. (2024). Bitter-tasting drugs tune GDF15 and GLP-1 expression via bitter taste or motilin receptors in the intestine of patients with obesity. Mol. Metab..

[12] Meyerhof W., Batram C., Kuhn C., Brockhoff A., Chudoba E., Bufe B., Appendino G., Behrens M. (2010). The molecular receptive ranges of human TAS2R bitter taste receptors. Chem. Senses.

[13] Behrens M., Gu M., Fan S.J., Huang C., Meyerhof W. (2018). Bitter substances from plants used in traditional Chinese medicine exert biased activation of human bitter taste receptors. Chem. Biol. Drug Des..

[14] Zeng B., Wei A.L., Zhou Q., Yuan M.H., Lei K.L., Liu Y.S., Song J.W., Guo L., Ye Q. (2022). Andrographolide: A review of its pharmacology, pharmacokinetics, toxicity and clinical trials and pharmaceutical researches. Phytother. Res..

[15] Song D.Y., Hao J.Y., Fan D.M. (2020). Biological properties and clinical applications of berberine. Front. Med..

[16] Wen Y.Q., Wang Y.Z., Zhao C.X., Zhao B.Y., Wang J.G. (2025). Baicalin: An active natural product with potential medicinal values. J. Asian Nat. Prod. Res..

[17] Hu X.L., Ao W.Z., Gao M.X., Wu L.J., Pei Y., Liu S.H., Wu Y.R., Zhao F., Sun Q.Q., Liu J.L. (2024). Bitter taste TAS2R14 activation by intracellular tastants and cholesterol. Nature.

[18] Xu W.X.., Wu L.J., Liu S.H., Liu X., Cao X.L., Zhou C., Zhang J.Y., Fu Y., Guo Y., Wu Y.R. (2022). Structural basis for strychnine activation of human bitter taste receptor TAS2R46. Science.

[19] Kim Y., Gumpper R.H., Liu Y.F., Kocak D.D., Xiong Y., Cao C., Deng Z.J., Krumm B.E., Jain M.K., Zhang S.C. (2024). Bitter taste receptor activation by cholesterol and an intracellular tastant. Nature.

[20] Wang X., Zhou C., Ao W.Z., Wu L.J., Wu Y.R., Xu W.X., Liu S.H., Tan Q.W., Wang L., Zhao F. (2025). Structural basis of β-glucopyranoside salicin recognition by a human bitter taste GPCR. Cell Rep..

[21] Page M.J., McKenzie J.E., Bossuyt P.M., Boutron I., Hoffmann T.C., Mulrow C.D., Shamseer L., Tetzlaff J.M., Akl E.A., Brennan S.E. (2021). The PRISMA 2020 statement: An updated guideline for reporting systematic reviews. BMJ.

[22] Zhang Y.X.., Wang X., Wang S.F., Zhang Y.L., Qiao Y.J. (2016). Traditional Chinese Bitter Flavor theory: Is there any relation with taste type II receptors?. Eur. J. Integr. Med..

[23] Tong A.Y., Yang H.Y., Yu X.H., Wang D.K., Guan J., Zhao M., Li J. (2025). Mechanisms and novel therapeutic roles of bitter taste receptors in diseases. Theranostics.

[24] Fan K.Q., Zhang L.M., Song F.Y., Zhang Y.H., Chen T., Cheng X., Su N., Zou Y., Yu T., Tan F.T. (2025). Pharmacological properties and therapeutic potential of berberine: A comprehensive review. Front. Pharmacol..

[25] Ren L., Ruan X.Q., Dong H.L., Cheng Y.Y., Shon K.Y., Chang C., Gu R.J., Sun Z.G. (2025). The bitter flavor of Banxia Xiexin decoction activates TAS2R38 to ameliorate low-grade inflammation in the duodenum of mice with functional dyspepsia. J. Ethnopharmacol..

[26] Jiao S.W., Yi H.L. (2026). OVA Inhalation and Baicalin Intervention: Unraveling Their Impact on Hepatic Function and the Involvement of Bitter Taste Signaling. J. Agric. Food Chem..

[27] Cui G.L., Wang M.L., Li X.F., Wang C., Shon K.Y., Liu Z.T., Ren L., Yang X.X., Li X.M., Wu Y.Y. (2024). Berberine in combination with evodiamine ameliorates gastroesophageal reflux disease through TAS2R38/TRPV1-mediated regulation of MAPK/NF-κB signaling pathways and macrophage polarization. Phytomedicine.

[28] Gervis J.E., Westerman K.E., Cole J.B., Merino J., Cromer S.J., Udler M.S. (2025). Functional variants in the TAS2R38 bitter taste receptor associate with postprandial glycemia. medRxiv.

[29] Meng X.H., Xi Z.C., Chen X.W., Green D., Zhou Y.H., Xian Y., Zhang H.M. (2025). Limonin: Advances in extraction, synthesis, pharmacological mechanisms, and structural optimization for therapeutic potential. Fitoterapia.

[30] De La Peña R., Hodgson H., Liu J.C., Stephenson M.J., Martin A.C., Owen C., Harkess A., Leebens-Mack J., Jimenez L.E., Osbourn A. (2023). Complex scaffold remodeling in plant triterpene biosynthesis. Science.

[31] Hu Y.X., Cai W., Zhang H.H., Yu C.H., Yu W.Y., Jin X.Y., Ying H.Z. (2016). Establishment and application of a model for screening natural active components with anti-asthma effects based on lung bitter taste receptors. Chin. Herb. Med..

[32] Tiroch J., Sterneder S., Di Pizio A., Lieder B., Hoelz K., Holik A.K., Pignitter M., Behrens M., Somoza M., Ley J.P. (2021). Bitter Sensing TAS2R50 Mediates the trans-Resveratrol-Induced Anti-inflammatory Effect on Interleukin 6 Release in HGF-1 Cells in Culture. J. Agric. Food Chem..

[33] Behrens M., Meyerhof W. (2011). Gustatory and extragustatory functions of mammalian taste receptors. Physiol. Behav..

[34] Sharma P., Conaway S., Deshpande D. (2022). Bitter Taste Receptors in the Airway Cells Functions. Handb. Exp. Pharmacol..

[35] Carey R.M., Palmer J.N., Adappa N.D., Lee R.J. (2023). Loss of CFTR function is associated with reduced bitter taste receptor-stimulated nitric oxide innate immune responses in nasal epithelial cells and macrophages. Front. Immunol..

[36] Stoeger V., Holik A.K., Hölz K., Dingjan T., Hans J., Ley J.P., Krammer G.E., Niv M.Y., Somoza M.M., Somoza V. (2020). Bitter-Tasting Amino Acids l-Arginine and l-Isoleucine Differentially Regulate Proton Secretion via T2R1 Signaling in Human Parietal Cells in Culture. J. Agric. Food Chem..

[37] Trius-Soler M., Moreno J.J. (2024). Bitter taste receptors: Key target to understand the effects of polyphenols on glucose and body weight homeostasis. Pathophysiological and pharmacological implications. Biochem. Pharmacol..

[38] Bloxham C.J., Foster S.R., Thomas W.G. (2020). A Bitter Taste in Your Heart. Front. Physiol..

[39] Foster S.R., Porrello E.R., Purdue B., Chan H.W., Voigt A., Frenzel S., Hannan R.D., Moritz K.M., Simmons D.G., Molenaar P. (2013). Expression, regulation and putative nutrient-sensing function of taste GPCRs in the heart. PLoS ONE.

[40] Lund T.C., Kobs A.J., Kramer A., Nyquist M., Kuroki M.T., Osborn J., Lidke D.S., Low-Nam S.T., Blazar B.R., Tolar J. (2013). Bone marrow stromal and vascular smooth muscle cells have chemosensory capacity via bitter taste receptor expression. PLoS ONE.

[41] Pensato V., Laginestra M.A., Falvo P., Orecchioni S., Talarico G., De Marchi E., Bruno S., Mongiorgi S., Mitola G., Bertolini F. (2024). Bitter Taste Receptor Agonist Denatonium Inhibits Stemness Characteristics in Hematopoietic Stem/Progenitor Cells. Stem Cells.

[42] Rudolph E., Dychtenberg H., Pozniak A., Pundir P. (2025). Bitter Taste Receptors in Bacterial Infections and Innate Immunity. Immun. Inflamm. Dis..

[43] Welcome M.O. (2020). The bitterness of genitourinary infections: Properties, ligands of genitourinary bitter taste receptors and mechanisms linking taste sensing to inflammatory processes in the genitourinary tract. Eur. J. Obstet. Gynecol. Reprod. Biol..

[44] Grădinaru T.C., Vlad A., Gilca M. (2023). Bitter phytochemicals as novel candidates for skin disease treatment. Curr. Issues Mol. Biol..

[45] Welcome M.O., Mastorakis N.E. (2021). The taste of neuroinflammation: Molecular mechanisms linking taste sensing to neuroinflammatory responses. Pharmacol. Res..

[46] Chen J., Larson E.D., Anderson C.B., Agarwal P., Frank D.N., Kinnamon S.C., Ramakrishnan V.R. (2019). Expression of Bitter Taste Receptors and Solitary Chemosensory Cell Markers in the Human Sinonasal Cavity. Chem. Senses.

[47] Carey R.M., Hariri B.M., Adappa N.D., Palmer J.N., Lee R.J. (2022). HSP90 Modulates T2R Bitter Taste Receptor Nitric Oxide Production and Innate Immune Responses in Human Airway Epithelial Cells and Macrophages. Cells.

[48] Yan C.H., Hahn S., McMahon D., Bonislawski D., Kennedy D.W., Adappa N.D., Palmer J.N., Jiang P., Lee R.J., Cohen N.A. (2017). Nitric oxide production is stimulated by bitter taste receptors ubiquitously expressed in the sinonasal cavity. Am. J. Rhinol. Allergy.

[49] Jalševac F., Descamps-Solà M., Grau-Bové C., Segú H., Auguet T., Avilés-Jurado F.X., Balaguer F., Jorba R., Beltrán-Debón R., Blay M.T. (2024). Profiling bitter taste receptors (TAS2R) along the gastrointestinal tract and their influence on enterohormone secretion. Gender- and age-related effects in the colon. Front. Endocrinol..

[50] Camillo L., Pollastro F., Talmon M., Fresu L.G. (2025). Bitter Taste Receptors 38 and 46 Regulate Intestinal Peristalsis. Int. J. Mol. Sci..

[51] Sternini C., Rozengurt E. (2025). Bitter taste receptors as sensors of gut luminal contents. Nat. Rev. Gastroenterol. Hepatol..

[52] Luo X.C., Chen Z.H., Xue J.B., Zhao D.X., Lu C., Li Y.H., Li S.M., Du Y.W., Liu Q., Wang P. (2019). Infection by the parasitic helminth Trichinella spiralis activates a Tas2r-mediated signaling pathway in intestinal tuft cells. Proc. Natl. Acad. Sci. USA.

[53] Wei X.Y., Lyu F.F., Jia F.Y., Zhang L.H., Zhang M., Xu Q., Hua S.Y. (2025). Bitter taste receptors in the gut-vascular axis: A novel target for immune and metabolic regulation of hypertension. Front. Immunol..

[54] Bloxham C.J., Hulme K.D., Fierro F., Fercher C., Pegg C.L., O’Brien S.L., Foster S.R., Short K.R., Furness S.G.B., Reichelt M.E. (2024). Cardiac human bitter taste receptors contain naturally occurring variants that alter function. Biochem. Pharmacol..

[55] Tsai C.C., Li Y.C., Chang L.C., Huang S.C. (2025). Bitter taste receptor agonists induce vasorelaxation in porcine coronary arteries. Front. Pharmacol..

[56] Malki A., Fiedler J., Fricke K., Ballweg I., Pfaffl M.W., Krautwurst D. (2015). Class I odorant receptors, TAS1R and TAS2R taste receptors, are markers for subpopulations of circulating leukocytes. J. Leukoc. Biol..

[57] Maurer S., Wabnitz G.H., Kahle N.A., Stegmaier S., Prior B., Giese T., Gaida M.M., Samstag Y., Hänsch G.M. (2015). Tasting Pseudomonas aeruginosa Biofilms: Human Neutrophils Express the Bitter Receptor T2R38 as Sensor for the Quorum Sensing Molecule N-(3-Oxododecanoyl)-l-Homoserine Lactone. Front. Immunol..

[58] Tran H.T.T., Herz C., Ruf P., Stetter R., Lamy E. (2018). Human T2R38 bitter taste receptor expression in resting and activated lymphocytes. Front. Immunol..

[59] Grassin-Delyle S., Salvator H., Mantov N., Abrial C., Brollo M., Faisy C., Naline E., Couderc L.J., Devillier P. (2019). Bitter taste receptors (TAS2Rs) in human lung macrophages: Receptor expression and inhibitory effects of TAS2R agonists. Front. Physiol..

[60] Talmon M., Camillo L., Vietti I., Pollastro F., Fresu L.G. (2024). Bitter taste receptor 46 (hTAS2R46) protects monocytes/macrophages from oxidative stress. Int. J. Mol. Sci..

[61] Deckmann K., Filipski K., Krasteva-Christ G., Fronius M., Althaus M., Rafiq A., Papadakis T., Renno L., Jurastow I., Wessels L. (2014). Bitter triggers acetylcholine release from polymodal urethral chemosensory cells and bladder reflexes. Proc. Natl. Acad. Sci. USA.

[62] Zhai K., Yang Z., Zhu X.F., Nyirimigabo E., Mi Y., Wang Y., Liu Q.H., Man L.B., Wu S.L., Jin J. (2016). Activation of bitter taste receptors (tas2rs) relaxes detrusor smooth muscle and suppresses overactive bladder symptoms. Oncotarget.

[63] Governini L., Semplici B., Pavone V., Crifasi L., Marrocco C., De Leo V., Arlt E., Gudermann T., Boekhoff I., Luddi A. (2020). Expression of taste receptor 2 subtypes in human testis and sperm. J. Clin. Med..

[64] Semplici B., Luongo F.P., Passaponti S., Landi C., Governini L., Morgante G., De Leo V., Piomboni P., Luddi A. (2021). Bitter taste receptors expression in human granulosa and cumulus cells: New perspectives in female fertility. Cells.

[65] Luongo F.P., Passaponti S., Haxhiu A., Raeispour M., Belmonte G., Governini L., Casarini L., Piomboni P., Luddi A. (2022). Bitter Taste Receptors and Endocrine Disruptors: Cellular and Molecular Insights from an In Vitro Model of Human Granulosa Cells. Int. J. Mol. Sci..

[66] Singh N., Vrontakis M., Parkinson F., Chelikani P. (2011). Functional bitter taste receptors are expressed in brain cells. Biochem. Biophys. Res. Commun..

[67] Duarte A.C., Santos J., Costa A.R., Ferreira C.L., Tomás J., Quintela T., Ishikawa H., Schwerk C., Schroten H., Ferrer I. (2020). Bitter taste receptors profiling in the human blood-cerebrospinal fluid-barrier. Biochem. Pharmacol..

[68] Roper S.D. (2007). Signal transduction and information processing in mammalian taste buds. Pflugers Arch..

[69] Kolesnikov S.S., Margolskee R.F. (1995). A cyclic-nucleotide-suppressible conductance activated by transducin in taste cells. Nature.

[70] Tuzim K., Korolczuk A. (2021). An update on extra-oral bitter taste receptors. J. Transl. Med..

[71] Richter P., Andersen G., Kahlenberg K., Mueller A.U., Pirkwieser P., Boger V., Somoza V. (2024). Sodium-Permeable Ion Channels TRPM4 and TRPM5 are Functional in Human Gastric Parietal Cells in Culture and Modulate the Cellular Response to Bitter-Tasting Food Constituents. J. Agric. Food Chem..

[72] Cheng W., Yao M.Y., Liu F.N. (2021). Bitter Taste Receptor as a Therapeutic Target in Orthopaedic Disorders. Drug Des. Devel. Ther..

[73] Talmon M., Rossi S., Lim D., Pollastro F., Palattella G., Ruffinatti F.A., Marotta P., Boldorini R., Genazzani A.A., Fresu L.G. (2019). Absinthin, an agonist of the bitter taste receptor hTAS2R46, uncovers an ER-to-mitochondria Ca(2+)-shuttling event. J. Biol. Chem..

[74] Talmon M., Massara E., Quaregna M., De Battisti M., Boccafoschi F., Lecchi G., Puppo F., Bettega Cajandab M.A., Salamone S., Bovio E. (2023). Bitter taste receptor (TAS2R) 46 in human skeletal muscle: Expression and activity. Front. Pharmacol..

[75] Cheng L., Xia F., Li Z.Y., Shen C.L., Yang Z.Q., Hou H.L., Sun S.Y., Feng Y.Y., Yong X.H., Tian X.W. (2023). Structure, function and drug discovery of GPCR signaling. Mol. Biomed..

[76] Brockhoff A., Behrens M., Massarotti A., Appendino G., Meyerhof W. (2007). Broad tuning of the human bitter taste receptor hTAS2R46 to various sesquiterpene lactones, clerodane and labdane diterpenoids, strychnine, and denatonium. J. Agric. Food Chem..

[77] Cannariato M., Fanunza R., Zizzi E.A., Miceli M., Di Benedetto G., Deriu M.A., Pallante L. (2024). Exploring TAS2R46 biomechanics through molecular dynamics and network analysis. Front. Mol. Biosci..

[78] Bartáková V., Kuricová K., Zlámal F., Bělobrádková J., Kaňková K. (2018). Differences in food intake and genetic variability in taste receptors between Czech pregnant women with and without gestational diabetes mellitus. Eur. J. Nutr..

[79] Carrai M., Steinke V., Vodicka P., Pardini B., Rahner N., Holinski-Feder E., Morak M., Schackert H.K., Görgens H., Stemmler S. (2011). Association between TAS2R38 gene polymorphisms and colorectal cancer risk: A case-control study in two independent populations of Caucasian origin. PLoS ONE.

[80] Yamaki M., Saito H., Isono K., Goto T., Shirakawa H., Shoji N., Satoh-Kuriwada S., Sasano T., Okada R., Kudoh K. (2017). Genotyping analysis of bitter-taste receptor genes TAS2R38 and TAS2R46 in Japanese patients with gastrointestinal cancers. J Nutr. Sci. Vitaminol..

[81] Choi J.H., Lee J., Choi I.J., Kim Y.W., Ryu K.W., Kim J. (2016). Genetic Variation in the TAS2R38 Bitter Taste Receptor and Gastric Cancer Risk in Koreans. Sci. Rep..

[82] Cantone E., Negri R., Roscetto E., Grassia R., Catania M.R., Capasso P., Maffei M., Soriano A.A., Leone C.A., Iengo M. (2018). In vivo biofilm formation, gram-negative infections and TAS2R38 polymorphisms in CRSw NP patients. Laryngoscope.

[83] Lee R.J., Xiong G., Kofonow J.M., Chen B., Lysenko A., Jiang P., Abraham V., Doghramji L., Adappa N.D., Palmer J.N. (2012). T2R38 taste receptor polymorphisms underlie susceptibility to upper respiratory infection. J. Clin. Invest..

[84] Dżaman K., Zagor M., Stachowiak M., Bielińska B., Sarnowska E., Krzeski A., Kantor I. (2016). The correlation of TAS2R38 gene variants with higher risk for chronic rhinosinusitis in Polish patients. Otolaryngol. Pol..

[85] Parsa S., Mogharab V., Ebrahimi M., Ahmadi S.R., Shahi B., Mehramiz N.J., Foroughian M., Zarenezhad M., Kalani N., Abdi M.H. (2021). COVID-19 as a worldwide selective event and bitter taste receptor polymorphisms: An ecological correlational study. Int. J. Biol. Macromol..

[86] Choi J.H., Lee J., Oh J.H., Chang H.J., Sohn D.K., Shin A., Kim J. (2017). Variations in the bitterness perception-related genes TAS2R38 and CA6 modify the risk for colorectal cancer in Koreans. Oncotarget.

[87] Lecchi G., Mocchetti C., Tunesi D., Berto A., Balasubramanian H.B., Biswas S., Bagchi A., Pollastro F., Fresu L.G., Talmon M. (2024). Single-nucleotide polymorphisms of TAS2R46 affect the receptor downstream calcium regulation in histamine-challenged cells. Cells.

[88] Talmon M., Bosso L., Quaregna M., Lopatriello A., Rossi S., Gavioli D., Marotta P., Caprioglio D., Boldorini R., Miggiano R. (2020). Anti-inflammatory Activity of Absinthin and Derivatives in Human Bronchoepithelial Cells. J. Nat. Prod..

[89] Shiffman D., O’Meara E.S., Bare L.A., Rowland C.M., Louie J.Z., Arellano A.R., Lumley T., Rice K., Iakoubova O., Luke M.M. (2008). Association of gene variants with incident myocardial infarction in the Cardiovascular Health Study. Arterioscler. Thromb. Vasc. Biol..

[90] Barontini J., Antinucci M., Tofanelli S., Cammalleri M., Dal Monte M., Gemignani F., Vodicka P., Marangoni R., Vodickova L., Kupcinskas J. (2017). Association between polymorphisms of TAS2R16 and susceptibility to colorectal cancer. BMC Gastroenterol..

[91] Choi J.H., Lee J., Yang S., Lee E.K., Hwangbo Y., Kim J. (2018). Genetic variations in TAS2R3 and TAS2R4 bitterness receptors modify papillary carcinoma risk and thyroid function in Korean females. Sci. Rep..

[92] Gentiluomo M., Crifasi L., Luddi A., Locci D., Barale R., Piomboni P., Campa D. (2017). Taste receptor polymorphisms and male infertility. Hum. Reprod..

[93] Zhang X., Ma J., Zhang A., Zhang J., Wu L., Wang F., Hu D. (2025). Bitter taste receptors as therapeutic targets: A review of the role and recent advances of bitter traditional Chinese medicine in bronchial asthma. J. Asthma.

[94] Costa A.R., Duarte A.C., Costa-Brito A.R., Gonçalves I., Santos C.R.A. (2023). Bitter taste signaling in cancer. Life Sci..

