# Supplementary material for: Bitter Chinese Herbal Medicine Exerts Pharmacological Effects via TAS2Rs: A Systematic Review from Natural Ligands to Therapeutic Potential
Source: Int J Mol Sci. 2026 Mar 27;27(7):3073. doi: 10.3390/ijms27073073 (PMC13073723; doi:10.3390/ijms27073073)
Supplement: Supplementary file 1 [file ijms-27-03073-s001.zip › ijms-4158656-supplementary.pdf]

Support material

Table S1. PRISMA 2020 Checklist.

Table S2. The expression, distribution, and physiological functions of TAS2Rs in the human body.

Table S3. Single nucleotide polymorphism (SNPs) in TAS2Rs and associated diseases.

Table S1. PRISMA 2020 Checklist.

Section and Topic	Item #	Checklist item	Location where item is reported
**TITLE**			
Title	1	Identify the report as a systematic review.	p.1, Title
**ABSTRACT**			
Abstract	2	See the PRISMA 2020 for Abstracts checklist.	p.1, Abstract
**INTRODUCTION**			
Rationale	3	Describe the rationale for the review in the context of existing knowledge.	p.1-2, Introduction
Objectives	4	Provide an explicit statement of the objective(s) or question(s) the review addresses.	p.3, Introduction, para 2 (Research Questions)
**METHODS**			
Eligibility criteria	5	Specify the inclusion and exclusion criteria for the review and how studies were grouped for the syntheses.	p.3, Section 2.1 and 2.2
Information sources	6	Specify all databases, registers, websites, organisations, reference lists and other sources searched or consulted to identify studies. Specify the date when each source was last searched or consulted.	p.3, Section 2.1 and 2.2
Search strategy	7	Present the full search strategies for all databases, registers and websites, including any filters and limits used.	p.3, Section 2.1 and 2.2
Selection process	8	Specify the methods used to decide whether a study met the inclusion criteria of the review, including how many reviewers screened each record and each report retrieved, whether they worked independently, and if applicable, details of automation tools used in the process.	p.3, Section 2.1 and 2.2
Data collection process	9	Specify the methods used to collect data from reports, including how many reviewers collected data from each report, whether they worked independently, any processes for obtaining or confirming data from study investigators, and if applicable, details of automation tools used in the process.	p.3, Section 2.2, para 1

Section and Topic	Item #	Checklist item	Location where item is reported
Data items	10a	List and define all outcomes for which data were sought. Specify whether all results that were compatible with each outcome domain in each study were sought (e.g. for all measures, time points, analyses), and if not, the methods used to decide which results to collect.	p.3, Section 2.
	10b	List and define all other variables for which data were sought (e.g. participant and intervention characteristics, funding sources). Describe any assumptions made about any missing or unclear information.	p.3, Section 2.2
Study risk of bias assessment	11	Specify the methods used to assess risk of bias in the included studies, including details of the tool(s) used, how many reviewers assessed each study and whether they worked independently, and if applicable, details of automation tools used in the process.	p.3, Section 2.2
Effect measures	12	Specify for each outcome the effect measure(s) (e.g. risk ratio, mean difference) used in the synthesis or presentation of results.	p.3, Section 2.2
Synthesis methods	13a	Describe the processes used to decide which studies were eligible for each synthesis (e.g. tabulating the study intervention characteristics and comparing against the planned groups for each synthesis (item #5)).	p.3, Section 2.2
	13b	Describe any methods required to prepare the data for presentation or synthesis, such as handling of missing summary statistics, or data conversions.	p.3, Section 2.2
	13c	Describe any methods used to tabulate or visually display results of individual studies and syntheses.	Results: Table S2 (p.9); Figures 1,2,3,4, 5 and 6 (p.20); Table S2
	13d	Describe any methods used to synthesize results and provide a rationale for the choice(s). If meta-analysis was performed, describe the model(s), method(s) to identify the presence and extent of statistical heterogeneity, and software package(s) used.	p.3, Section 2.2
	13e	Describe any methods used to explore possible causes of heterogeneity among study results (e.g. subgroup analysis, meta-regression).	p.3, Section 2.2
	13f	Describe any sensitivity analyses conducted to assess robustness of the synthesized results.	p.5, Section 2.2
Reporting bias	14	Describe any methods used to assess risk of bias due to missing results in a synthesis (arising from reporting biases).	p.5, Section 2.2

Section and Topic	Item #	Checklist item	Location where item is reported
assessment			
Certainty assessment	15	Describe any methods used to assess certainty (or confidence) in the body of evidence for an outcome.	no
**RESULTS**			
Study selection	16a	Describe the results of the search and selection process, from the number of records identified in the search to the number of studies included in the review, ideally using a flow diagram.	p.3, Figure 1
	16b	Cite studies that might appear to meet the inclusion criteria, but which were excluded, and explain why they were excluded.	p.3, Section 2. 1
Study characteristics	17	Cite each included study and present its characteristics.	Table S2 and S3
Risk of bias in studies	18	Present assessments of risk of bias for each included study.	no
Results of individual studies	19	For all outcomes, present, for each study: (a) summary statistics for each group (where appropriate) and (b) an effect estimate and its precision (e.g. confidence/credible interval), ideally using structured tables or plots.	p.3-13, Table S2 and S3
Results of syntheses	20a	For each synthesis, briefly summarise the characteristics and risk of bias among contributing studies.	p.3-13
	20b	Present results of all statistical syntheses conducted. If meta-analysis was done, present for each the summary estimate and its precision (e.g. confidence/credible interval) and measures of statistical heterogeneity. If comparing groups, describe the direction of the effect.	p.3-13
	20c	Present results of all investigations of possible causes of heterogeneity among study results.	p.3-13
	20d	Present results of all sensitivity analyses conducted to assess the robustness of the synthesized results.	p.3-13
Reporting biases	21	Present assessments of risk of bias due to missing results (arising from reporting biases) for each synthesis assessed.	p.3-13

Section and Topic	Item #	Checklist item	Location where item is reported
Certainty of evidence	22	Present assessments of certainty (or confidence) in the body of evidence for each outcome assessed.	p.3-13
**DISCUSSION**			
Discussion	23a	Provide a general interpretation of the results in the context of other evidence.	p.13-14
	23b	Discuss any limitations of the evidence included in the review.	p.13-14
	23c	Discuss any limitations of the review processes used.	p.13-14
	23d	Discuss implications of the results for practice, policy, and future research.	p.13-14
**OTHER INFORMATION**			
Registration and protocol	24a	Provide registration information for the review, including register name and registration number, or state that the review was not registered.	p.2
	24b	Indicate where the review protocol can be accessed, or state that a protocol was not prepared.	p.2
	24c	Describe and explain any amendments to information provided at registration or in the protocol.	p.2
Support	25	Describe sources of financial or non-financial support for the review, and the role of the funders or sponsors in the review.	p.14
Competing interests	26	Declare any competing interests of review authors.	p.14
Availability of data, code and other materials	27	Report which of the following are publicly available and where they can be found: template data collection forms; data extracted from included studies; data used for all analyses; analytic code; any other materials used in the review.	p.14

From: Page, M.J.; McKenzie, J.E.; Bossuyt, P.M.; Boutron, I.; Hoffmann, T.C.; Mulrow, C.D.; Shamseer, L.; Tetzlaff, J.M.; Akl, E.A.; Brennan, S.E.; et al. The PRISMA 2020 statement: An updated guideline for reporting systematic reviews. *BMJ* **2021**, *372*, n71. [21]

Table S2 The expressions, distributions, and physiological functions of TAS2Rs in human tissues

System	Distributions	Subtypes	The main regulatory mechanism	Pharmacology	References
Respiratory system					
	Solitary chemosensory cells (SCCs)	TAS2R4/14/46	Via the canonical PLCβ2-IP3 signaling pathway	Neurogenic inflammation airway immune defense	[30]
	Nasal epithelial cells (NECs)	TAS2R4/16/38	Via the canonical PLCβ2-IP3 signaling pathway	Antibacterial effects	[31]
	Airway ciliated (epithelial) cells	TAS2R4/38/43/46	Increase in cytosolic Ca^{2+} concentration is mediated by the canonical PLCβ2-IP3 signaling pathway	Clear foreign microorganisms and their metabolic products	[32]
	Airway Smooth Muscle	TAS2R10/14/31/5/4/19/3/20/45/50/30/9/13/42/46/1/8	Activation of the canonical PLCβ2-IP3 pathway by generating localized Ca^{2+} signals that stimulate BKCa channels.	Elicits membrane hyperpolarization	[41]
	Bronchial Smooth Muscle	TAS2R5/10/14	PI3K may negatively regulate TAS2Rs	Induced relaxation	[42]
Digestive system					
	Gastric Mucosa (Parietal Cell, Chief Cell）	TAS2R7/10/14/43/46	Activation of the adenylate cyclase (AC)-cAMP pathway	Activates the proton pump in gastric parietal cells, thereby stimulating gastric acid secretion.	[10]
	Gastric Smooth Muscle Cell	TAS2R3/4/10	Activation of the canonical PLCβ2-IP3 pathway by low-dose agonists, via a PKC-	Induces smooth muscle contraction; induce	[44]

			dependent mechanism by high concentrations	relaxation or inhibit contraction	
	Paneth cells and goblet cells in the jejunal crypts	TAS2R10/43	TAS2R10 releases antimicrobial peptides such as defensins and lysozymes. TAS2R43 is a dependent genetic polymorphism	Destroy the structure of bacteria with TAS2R10. regulates the secretion of mucin proteins CLCA1 and MUC2 with TAS2R43.	[43]
	HuTu-80 and NCI-H716 cells	TAS2R3/4/5/10/13/38/39/40/42/43/44/45/46/47/49/50/60	Through the Gαgust-dependent signaling pathway	May regulate the secretion of PYY and GLP-1.	[33]
	Small Intestine and Pancreas	TAS2R14	-	-	[22]
Cardiovascular system					
	Myocardial Tissue (Cardiomyocyte and Fibroblast)	TAS2R3/4/5/9/10/13/14/19/20/30/31/43/45/46/50	Through the PLCβ2-IP3 signaling pathway	Regulate cardiac metabolism	[34]
	Vascular Smooth Muscle Cell	TAS2R46	Activate cAMP-ATP and PLCβ2-IP3 signaling pathways.	Dilate blood vessels.	[35]
Immune system					
	Leukocyte	All TAS2Rs	Through the classical PLCβ2-IP3 signaling pathway.	Induction of neutrophil chemotaxis and migration	[36]
	Neutrophil	TAS2R38	Enhances neutrophil chemotaxis and phagocytosis, and upregulates the surface adhesion receptor CD11b	Boosting cellular adhesion and migratory capacity	[46]

	CD34⁺ Hematopoietic stem cells, monocytes, macrophages, neutrophils, osteoclasts	TAS2R38	Recruitment of IQGAP1 by the G protein-dependent pathway, coordinated with calcium signaling and p38 MAPK activation	Potentiates phagocytosis, cell adhesion, and antiviral defense	[91]
	Lymphocyte	TAS2R38	Release Ca^{2+} from endoplasmic reticulum stores by the PLCβ2-IP3 signaling pathway	Anti-inflammation	[47]
	Pulmonary Macrophage	TAS2R3/4/5/7/8/9/10/14/19/20/31/38/39/43/45/46	Inhibit the release of inflammatory factors such as TNF-α, CCL3, and CXCL8	Anti-inflammation	[48]
	Macrophages, Monocytes	TAS2R10/14/30/46	Attenuate PMA-induced oxidative stress by reducing ROS and RNS levels	Regulates oxidative stress	[49]
Urinary system					
	Detrusor muscle	TAS2R1/4/5/7/8/9/10/13/14/20/30/31/38/39/40/45/50	-	-	[51]
Genital system					
	Testis and Sperm	TAS2R3/4/14/19/43	Via the PDE-cAMP and PLCβ2-IP3 signaling pathways	Triggers membrane depolarization	[52]
	Granulosa Cells and Cumulus Cells	TAS2R3/4/14/19/43	Via the PDE-cAMP and PLCβ2-IP3 signaling pathways	Regulate the follicular microenvironment and hormone synthesis	[53]
	Granulosa Cell	TAS2R3/4/14/19/43	Regulates mitochondrial dynamics and induces remodeling of lipid droplets.	Affecting the efficiency of steroid synthesis and the	[54]

				utilization rate of cholesterol	
	Uterine Tissue	TAS2R1/4/10/14/38	-	-	[55]
	Syncytiotrophoblas and amniotic epithelium	TAS2R38	Via G protein-dependent calcium signaling pathway	-	[56]
	Placental Trophoblas, Villous Tissue and Basal Plate	TAS2R14	Via G protein-dependent calcium signaling pathway	-	[57]
	Brain cells	TAS2R4/10/38	Activate the G protein-dependent calcium signaling pathway	-	[58]
Nervous system	Epithelial cells of the choroid plexus	TAS2R1/3/4/5/7/8/9/13/14/16/38/39/41/42/43/44/45/46/48/49/50/60	-	-	[59]

Note: -, it indicates that the relevant regulatory mechanisms of TAS2Rs in human tissues are not mentioned in the related article.

Table S3 SNPs in TAS2Rs gene and its disease relevance.

TAS2Rs	Characteristics of SNP	Related diseases	Source of the research population	Genotype analysis	Analysis of disease correlation	References
TAS2R38	rs713598 (G145C, A49P）, rs1726866(T785C, V262A), rs10246939(A886G ,I296V)	Upper respiratory infection	-	The PAV/PAV genotype was absent in patients infected with Gram-negative bacteria. In vitro exposure of Pseudomonas aeruginosa to PAV/PAV genotype epithelial cells resulted in a markedly reduced bacterial survival rate of only about 14%.	The presence of the PAV gene enhances intracellular Ca^{2+} influx, promoting the production of NO, enhancing ciliary movement and mucus clearance, and exerting a direct bactericidal effect.	[77]
		Chronic nasosinusitis	Poland	The AVI/PAV type has the highest occurrence rate (50%), and the frequency of PAV/PAV is the lowest (22%).	Different polymorphisms have different effects on the susceptibility to chronic sinusitis.	[78]
		Chronic nasosinusitis	Italy	The VI/AVI double type is more common in patients with chronic sinusitis (25%). The AVI/AVI and AVI/PAV types together account for 88.9%, while the PAV/PAV type accounts for only 11.1% (P = 0.023). In the AVI/AVI double type, the formation of biofilms is widespread (62.5%, P = 0.05).	The AVI haplotype is strongly associated with airway Gram-negative bacterial infections, increasing the susceptibility to chronic sinusitis and being related to the formation of extracellular biofilms.	[76]
		Primary ciliary dyskinesia	Italy	The prevalence rate of PAV/PAV type was 28.6%, while the prevalence rates of PAV/AVI and AVI/AVI types were	The PAV/PAV double mutant has a Potential protective effect in primary ciliary	[92]

		48.6% and 22.8%, respectively.	dyskinesia.	
Primary ciliary dyskinesia	Italy	(For the DNAH11 mutation group) Compared with patients with AVI/AVI type, the nNO level of patients with PAV/PAV type was significantly higher ($p = 0.033$), and the chronic infection rate of patients carrying the PAV allele (PAV/PAV or PAV/AVI) was significantly lower ($P = 0.029$).	It may act as a modifier gene for the severity of primary ciliary dyskinesia, but it is only significant in patients with milder DNAH11 mutations.	[93]
COVID-19	22 different countries	PAV /AVI ($r = -0.572$, $p = 0.021$)	The PAV haplotype may play a protective role in the innate immunity of the upper respiratory tract mucosa.	[79]
CColorectal cancer	Czechia and Germany	PAV/AVI：<br>OR=1.00, 95%CI=0.79-1.27, p=0.948<br>AVI/AVI：<br>OR=1.33, 95%CI=1.03-1.72, p=0.027	The AVI/AVI homozygous diploid type significantly increases the risk of colorectal cancer.	[94]
Gastrointestinal cancer	Japan	AVI/AVI：<br>OR=2.04, 95%CI=1.095-3.815, p=0.024<br>AVI/PAV:<br>OR=1.07, 95%Cl=0.630-1.806, p=0.811<br>PAV/PAV：<br>OR=0.55, 95%CI=0.299-0.996, p=0.048	The AVI/AVI homozygous diplotype significantly increases the risk for gastrointestinal cancers, whereas the PAV/PAV diplotype is a protective genotype associated with lower cancer risk.	[74]

		Gastric cancer	Korea	PAV/AVI：<br>OR=1.513, 95%CI=1.148-1.994, p=0.001<br>AVI/AVI：<br>OR=0.956, 95%CI=0.656-1.394, p=0.145	The presence of PAV/AVI heterozygotes increases the risk of gastric cancer.	[75]
		Colorectal cancer	Korea	PAV/AVI：<br>OR=0.94, 95%CI=0.76-1.18, p=0.600<br>AVI/AVI：<br>OR=0.70;95%Cl=0.51-0.95, p=0.022<br>When the CA6-G allele is present, it enhances the AVI/AVI protective effect. OR=0.49, 95%CI=0.34-0.74, p<0.001）	AVI/AVI reduces the susceptibility to colorectal cancer.	[80]
	rs72477411(-, I147V)			Significantly reduces mitochondrial calcium uptake		
	rs72477410(-, I153V)			Inhibits the mitochondrial calcium regulatory pathway, resulting in the complete abolition of mitochondrial calcium uptake.		
TAS2R50	rs1376251(C/T, C203Y)	Myocardial infarction	White elderly	Increases the risk of white people contracting diseases by 13-14% (HR=1.14, 90%CI=1.01-1.28, p=0.038)	This gene may have other variations related to myocardial infarction.	[46, 84]
TAS2R16	rs1525489(T/C, -)	Colorectal cancer	Europe	There was no significant association with overall colorectal cancer risk, while the allele C increased the risk of rectal cancer (P=0.0071).	The association of allele C with an increased risk of rectal cancer was not significant.	[84]

TAS2R4	rs2234001(C/G, V96L)	Papillary thyroid carcinoma	Korea	Significantly reduces the risk of papillary thyroid carcinoma by decreasing total triiodothyronine (TT3) levels (OR=0.59;95%CI=0.36-0.97, p=0.036).	The C/C haplotype can reduce the risk of PTC.	[85]
TAS2R3	rs2270009(T/C, -)					
	rs11763979(G/T，-)	Male infertility	Europe	Significantly lower in carriers of the T/T homozygous genotype than in G/G homozygous carriers (P=0.002).	Potentially leading to reduced WEE2 protein levels and compromised sperm morphology.	[86]
TAS2R14	rs3741843(A/G, -)	Male infertility	Europe	Significantly lower in carriers of the G/G homozygous genotype than in A/A homozygous carriers (P=0.003).	May affect the flagellar movement of sperm.	[86]

Note: T/C. T represents the main allele, while C is the secondary allele (the specific location of the base mutation is not indicated in the literature. -, indicating that the amino acids have not changed
